# Heartwood of *Dalbergia cochinchinensis*: 4,7,2′-Trihydroxy-4′-methoxyisoflavanol and 6,4′-Dihydroxy-7-methoxyflavane Reduce Cytokine and Chemokine Expression In Vitro

**DOI:** 10.3390/molecules27041321

**Published:** 2022-02-15

**Authors:** Feng Shao, Layla Panahipour, Mariane Beatriz Sordi, Fangrui Tang, Ronghua Liu, Reinhard Gruber

**Affiliations:** 1 Department of Oral Biology, Medical University of Vienna, 1090 Vienna, Austria; layla.panahipour@meduniwien.ac.at (L.P.); marianesordi@hotmail.com (M.B.S.); 2 Key Laboratory of Modern Preparation of Traditional Chinese Medicine, Ministry of Education, Jiangxi University of Chinese Medicine, Nanchang 330004, China; tangfangrui@163.com (F.T.); rhliu@163.com (R.L.); 3 Key Laboratory of Innovation Drug and Efficient Energy-Saving Pharmaceutical Equipment, Jiangxi University of Chinese Medicine, Nanchang 330004, China; 4 Department of Dentistry, Federal University of Santa Catarina, Florianopolis 88040-900, Brazil; 5 Department of Periodontology, School of Dental Medicine, University of Bern, 3012 Bern, Switzerland; 6 Austrian Cluster for Tissue Regeneration, 1200 Vienna, Austria

**Keywords:** flavonoids, *Dalbergia cochinchinensis*, macrophages, fibroblasts

## Abstract

*Dalbergia cochinchinensis* has been widely used in traditional medicine because of its flavonoids; however, the impact of the flavonoids to modulate the inflammatory response to oral cells remains to be described. For this aim, we isolated 4,7,2′-trihydroxy-4′-methoxyisoflavanol (472T4MIF) and 6,4′-dihydroxy-7-methoxyflavane (64D7MF) from the heartwood of *D. cochinchinensis* and confirmed the chemical structure by nuclear magnetic resonance. We show here that both flavonoids are inhibitors of an inflammatory response of murine RAW 264.7 inflammatory macrophages stimulated by LPS. This is indicated by interleukin (IL)1, IL6, and chemokine CCL2 production besides the phosphorylation of p65. Consistently, in primary murine macrophages, both flavonoids decreased the inflammatory response by lowering LPS-induced IL1 and IL6 expression. To introduce oral cells, we have used human gingival fibroblasts and provoked the inflammatory response by exposing them to IL1β and TNFα. Under these conditions, 472T4MIF, but not 64D7MF, reduced the expression of chemokines CXCL1 and CXCL2. Taken together, we identified two flavonoids that can reduce the expression of cytokines and chemokines in macrophages and fibroblastic cells.

## 1. Introduction

*Dalbergia cochinchinensis* is a species of legumein of the family *Fabaceae*, which is widely distributed in Southeast Asia, particularly in Cambodia, Lao PDR, Thailand, and Vietnam. Its drought tolerance and nitrogen-fixing ability make *D. cochinchinensis* a deciduous tree in agroforestry systems of the tropics, particularly for restoring degraded forests and deforested sites [1]. However, *Dalbergia* is highly threatened as a genus globally because of its deforestation and illegal harvesting, with *D. cochinchinensis* characterized as vulnerable and engendered in the IUCN Red List of Threatened Species™. There is nevertheless a need for research to understand the biology of *D*. *cochinchinensis*, and natural plant-derived products have been recognized as one of the main sources for drug discovery. 

The transcriptomes of *D. cochinchinensis* and other species were deciphered [2,3,4]. Moreover, the stress response of *D. cochinchinensis* to drought and heat was reported [1], and the CO_2_ binding capacity was assessed [5]. Volatile components in *D. cochinchinensis* were also analysed [6]. Chemical constituents from the heartwood of *D. cochinchinensis* were isolated [7,8,9], e.g., 6,4′-dihydroxy-7-methoxyflavan [9]. From a clinical perspective, occupational toxic epidermal necrolysis was associated with *D. cochinchinensis* [10], and some compounds showed inhibitory activity towards 5α-dihydrotestosterone [9].The impact of isolated compounds of *D. cochinchinensis* to modulate an inflammatory response is rather unknown. 

Most research has been done with *D. odorifera*. Lipopolysaccharide(LPS)-stimulated RAW 264.7 cells were used to identify phytocompounds isolated from *D. odorifera* to show an activity [11,12]. Additionally, latifolin, a neoflavonoid extracted from *D. odorifera,* is capable to protect against myocardial infarction by targeting IL6 signalling [13] and cardiotoxicity including macrophage polarization [14]. 6,7,4′-Trihydroxyflavone from *D. odorifera* also prevented LPS-induced bone loss in a mice model [15]. Consistently, the effect of *D. odorifera* extract was confirmed by LPS intraperitoneal injection in a mouse model and in vitro with the murine RAW 264.7 macrophage cell line [16]. 9-Hydroxy-6,7-dimethoxydalbergiquinol and 6,4′-dihydroxy-7-methoxyflavanone isolated from the heartwood of *D. odorifera* prevent osteoclast differentiation [17,18]. Sativanone from *D. odorifera* also inhibits nitric oxide and tumour necrosis factor-alpha (TNFα) release from LPS-stimulated RAW 264.7 cells [19]. Nevertheless, similar research with phytochemicals isolated from *D. cochinchinensis* has not been done so far. 

RAW 264.7 cells, primary macrophages, and gingival fibroblasts, particularly in the dental field [20], are widely used to test if a candidate molecule can change the expression of cytokines and chemokines that are key mediators of an inflammatory reaction. The inflammatory environment in vitro is simulated by exposing macrophages to bacterial-derived virulence factors such as LPS, which activates the Toll-like receptor-4 (TLR4)signalling. This pathway then drives the expression of interleukin 1 (IL1), IL6, and chemokines including chemokine (C-C motif) ligand 2 (CCL2), also referred to as monocyte chemoattractant protein 1, all of which are involved in chronic inflammation, such as periodontitis [21]. For instance, CCL2 exhibits a chemotactic activity for monocytes and basophils and thus is implicated in the pathogenesis of several diseases characterized by cell infiltrates. Gingival fibroblasts exposed to inflammatory cytokines IL1β and TNFα also serve as an in vitro model to study how a candidate molecule lowers inflammatory reactions indicated by the expression of chemokines CXCL1 and CXCL2 [22]. These and other chemokines attract immune cells, especially neutrophils and hematopoietic progenitors, for instance, in the rheumatoid arthritis [23]. The in vitro models do not simulate the complexity of an inflammatory reaction in vivo but are resourceful tools to identify phytochemicals with a possible anti-inflammatory activity. These candidate molecules may then undergo preclinical testing considering the complexity of in vivo inflammation onset and its resolution. 

Here, we have identified 4,7,2′-trihydroxy-4′-methoxyisoflavanol (472T4MIF) and 6,4′-dihydroxy-7-methoxyflavane (64D7MF) to be inhibitors of an in vitro inflammatory response with the overall aim to identify natural compounds and support their use in herbal medicine.

## 2. Material and Methods

### 2.1. Extraction of Compounds

The isolation of the compounds was reported in detail [24,25]. In brief, the heartwood of *D. cochinchinensis* was kindly provided by Prof. Yingjian Li, Guangxi University, Nanning, China. A voucher specimen was deposited in the Key Laboratory of Modern Preparation of TCM, Ministry of Education, Jiangxi University of Chinese Medicine. The air-dried heartwood of *D. cochinchinensis* (20.0 kg) was powdered, passed through a #40 mesh sieve, and extracted with 70% ethanol under refluxing three times, for 2 h. After removing the solvent under reduced pressure, the residue (5.4 kg) was suspended in water and partitioned with petroleum ether (60~90 °C), chloromethane, ethyl acetate, and n-butanol, respectively. 

### 2.2. Isolation of 4,7,2′-Trihydroxy-4′-methoxyisoflavanol (472T4MIF)

The petroleum ether extract (381.2 g) was separated into nine fractions (Frs. 1–9) by applying a silica gel column, eluted with petroleum ether-EtOAc (50:1 to 1:1). Fr. 7 (6.0 g) was separated into ten fractions (Frs. 7A–7J) by applying a silica gel column, eluted with petroleum ether- CH_2_Cl_2_ (2:1 to 1:2). Fr. 7I (201.3 mg) was purified by Sephadex LH-20 with CH_2_Cl_2_-MeOH (1:1) to give 472T4MIF (139.8 mg). The chemical structure of 472T4MIF was confirmed by nuclear magnetic resonance spectroscopy [26].472T4MIF was dissolved in dimethyl sulfoxide and stored in aliquots until being used for the in vitro testing.

### 2.3. Isolation of 6,4′-Dihydroxy-7-methoxyflavane (64D7MF)

The chloroform extract (902.6 g) was separated into 9 fractions (Frs. 1–9) by adopting a silica gel column and was eluted with a gradient mixture of petroleum ether-EtOAc (50:1 to 1:1). Fr. 5 was separated over silica gel with CH_2_Cl_2_-MeOH (50:1 to 1:1) and was purified by Sephadex LH-20 using CH_2_Cl_2_-MeOH (1:1) as an eluent to afford 64D7MF (576.4 mg). The chemical structures of 64D7MFwere confirmed by nuclear magnetic resonance spectroscopy [9]. They were dissolved in dimethyl sulfoxide and stored in aliquots until being used for the in vitro testing.

### 2.4. Murine RAW 264.7 Cells, Primary Macrophages, and Human Gingival Fibroblasts

RAW 264.7 macrophage-like cells (ATCC; LGC Standards GmbH, Wesel, Germany) were expanded in regular Dulbecco’s Modified Eagle Medium (DMEM) with 10% fetal calf serum (FCS) and 1% of 10,000 units penicillin and 10 mg streptomycin/mL (PS) (Sigma, St Louis, MO, USA), and seeded at 3 × 10^5^/cm^2^ for the experiments. BALB/c mice at the age of 6–8 weeks were purchased from Animal Research Laboratories, Himberg, Austria. The femora and tibiae of the mice were removed after scarifying, and bone marrow cells were collected. Bone marrow cells were seeded at 1 × 10^6^ cells/cm^2^ into 12-well plates and grown for 5–7 days in DMEM supplemented with 10% FCS and 1% PS (Sigma, St Louis, MO, USA) and with 20 ng/mL mouse macrophage colony-stimulating factor (M-CSF; ProSpec-Tany Techno Gene Ltd., Rehovot, Israel). Human gingival fibroblasts were prepared by explant cultures after approval of the Ethical Committee of the Medical University of Vienna (EK Nr. 631/2007). Gingival fibroblasts were expanded in DMEM supplemented with 10% FCS and 1% PS (Sigma, St Louis, MO, USA) and seeded at 3 × 10^5^/cm^2^. All cells were cultured under standard conditions of 37 °C, 5% CO_2_, and 95% humidity.

### 2.5. Cell Stimulation

Primary macrophages and RAW 264.7 cells were exposed to 20 µM 4,7,2′-trihydroxy-4′-methoxyisoflavanol and 6,4′-dihydroxy-7-methoxyflavane for 30 min before adding 100 ng/mL LPS for 24 h. Gingival fibroblasts were exposed to both flavonoids for 30 min before being challenged by IL1β and TNFα (both at 10 ng/mL, ProSpec) in serum-free DMEM. After 24 h, gene expression analysis was performed, and the supernatant was collected for immunoassays.

### 2.6. RT-PCR and Immunoassay

Total RNA was isolated with the ExtractMe total RNA kit (Blirt S.A., Gdańsk, Poland). Complementary DNA was synthesized through reverse transcription of the total RNA (LabQ, Labconsulting, Vienna, Austria), and a polymerase chain reaction was performed with a master mix from LabQ (Labconsulting, Vienna, Austria). Amplification was monitored on the CFX Connect™ Real-Time PCR Detection System (Bio-Rad Laboratories, Hercules, CA, USA). Primer sequences are provided in Table 1. The mRNA levels were calculated by normalization to the housekeeping gene GAPDH using the ΔΔCt method. For the immunoassay, the mouse IL6 kit was used (R&D Systems, Minneapolis, MN, USA).

### 2.7. Western Blot

RAW 264.7 cells and gingival fibroblasts were serum-starved overnight and then preincubated for 30 min with the flavonoids before being exposed for 30 min to LPS (100 ng/mL). Cell extracts containing SDS buffer and protease inhibitors (PhosSTOP with complete; Sigma, St. Louis, MO, USA) were separated by SDS-PAGE and transferred onto polyvinylidene fluoride (PVDF) membranes (Roche Diagnostics, Mannheim, Germany). Membranes were blocked and the binding of the first antibody raised against phosphorylated and non-phosphorylated p65 and p38 (Cell Signaling Technology, Danvers, MA, USA), and β-actin (Santa Cruz Biotechnology, Santa Cruz, CA, USA) was detected with the appropriate secondary antibody linked to a peroxidase. Chemiluminescence signals were visualized with the ChemiDoc imaging system (Bio-Rad Laboratories, Inc., Hercules, CA, USA).

### 2.8. Statistical Analysis

All experiments were repeated at least three times. Data from individual experiments are shown as dot-blots. For PCR, data are described as an x-fold change compared to the unstimulated control. Statistical analysis was based on repeated measures of one-way ANOVA with Greehouse–Geisser correction and Dunnett’s multiple comparison test. The *p*-values are indicated compared to the stimulated group but without the flavonoids. Significance was set at *p* < 0.05. Data were analysed by the Prism 8.0e software (GraphPad Software; San Diego, CA, USA).

## 3. Results

### 3.1. Isolation of 472T4MIF and 64D7MF

**Figure 1:**
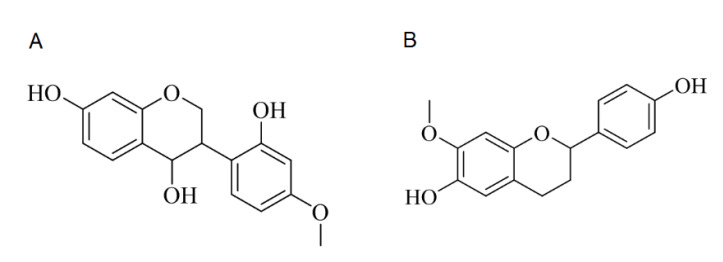
The structures of flavonoids. The ethanolic extract of the heartwood of *D. cochinchinensis* was further separated allowing the isolation of (**A**) 472T4MIF and (**B**) 64D7MF. Their chemical structures were confirmed by nuclear magnetic resonance spectroscopy.

To isolate chemically defined natural compounds, we reconstituted the ethanolic extracts of the heartwood of *D. cochinchinensis* with petroleum ether, chloromethane, ethyl acetate, n-butanol dichloromethane and ethyl acetate. The petroleum ether extract (381.2 g) was further separated isolate 472T4MIF (139.8 mg, Figure 1A). The chloroform extract (902.6 g) was separated and finally 64D7MF (576.4 mg) was isolated (Figure 1B). The chemical structures of 472T4MIF and 64D7MF were confirmed by nuclear magnetic resonance spectroscopy. They were dissolved in dimethyl sulfoxide and stored in aliquots until being used for the in vitro testing.

### 3.2. 472T4MIF and 64D7MF Do Not Affect Viability of RAW 264.7 Macrophages

**Figure 2:**
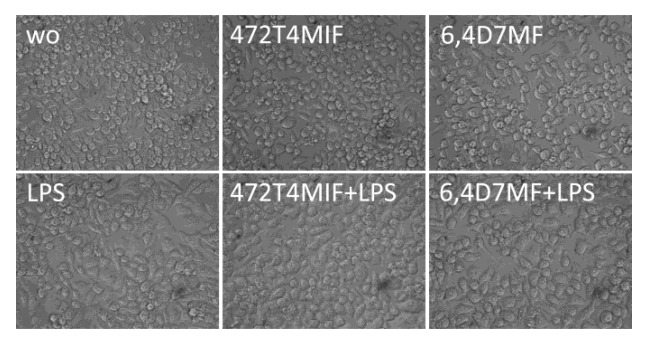
Morphology of RAW 264.7 cells exposed to 472T4MIF and 64D7MF and LPS.RAW 264.7 cells were exposed to 20 µM of 472T4MIF or 64D7MF for 30 min before cells were treated with 100 ng/mL LPS for 24 h. WO indicates unstimulated control. The phase-contrast images indicate the LPS supports cell attachment but 472T4MIF and 64D7MF caused no obvious deterioration of cell morphology.

To exclude the possible toxicity of 472T4MIF and 64D7MF, we grew RAW 264.7 macrophages in the presence of the two flavonoids before provoking an inflammatory response with LPS [27,28]. We report that there were no morphological changes of cells in phase-contrast microscopy (Figure 2). Moreover, the conversion of a substrate into formazan crystals was not affected by 20 µM 472T4MIF and 64D7MF alone or in the presence of LPS (data not shown). These findings indicate that RAW 264.7 macrophages remain viable when exposed to the indicated concentration of 472T4MIF and 64D7MF.

### 3.3. 472T4MIF and 64D7MF Have an Activity in Murine Macrophages

**Figure 3:**
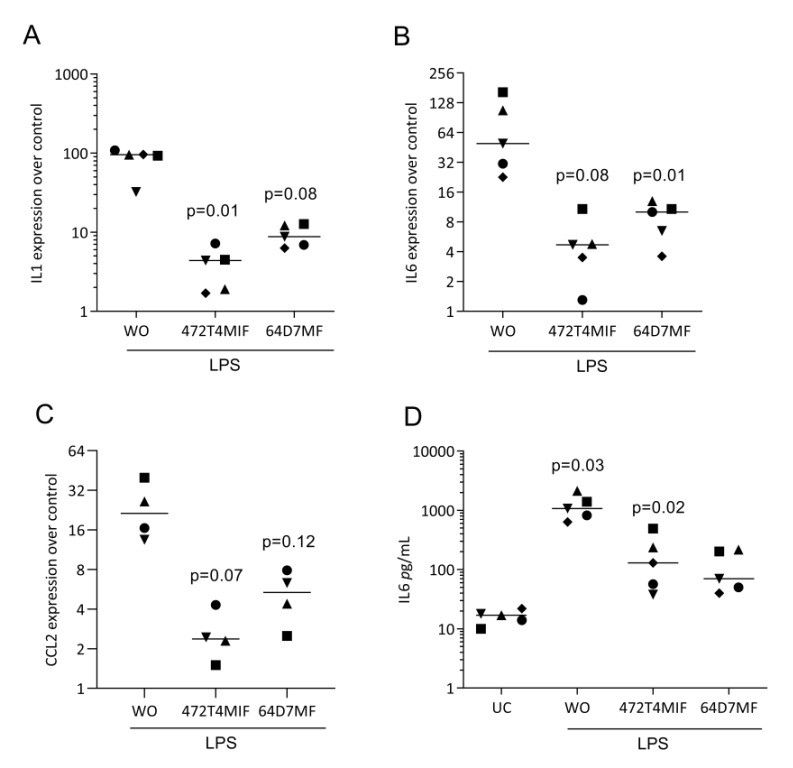
472T4MIF and 64D7MF suppress LPS-induced expression of cytokines in RAW 264.7 cells. RAW 264.7 cells were exposed to 20 µM of 472T4MIF or 64D7MF for 30 min before cells were treated with 100 ng/ml LPS for 24 h. WO indicates LPS but no flavonoids. Data show the x-fold changes in (**A**) IL1, (**B**) IL6, and (**C**) CCL2 expression compared to unstimulated cells (expression of control which is 1). (**D**) The immunoassay shows the release of IL6 into the supernatant of the respective cultures with unstimulated cells (UC) indicating cells without LPS. Statistical analysis was based on repeated measures one-way ANOVA with Greenhouse–Geisser correction and Dunnett’s multiple comparison test. We compared the LPS group to the other groups.

**Figure 4:**
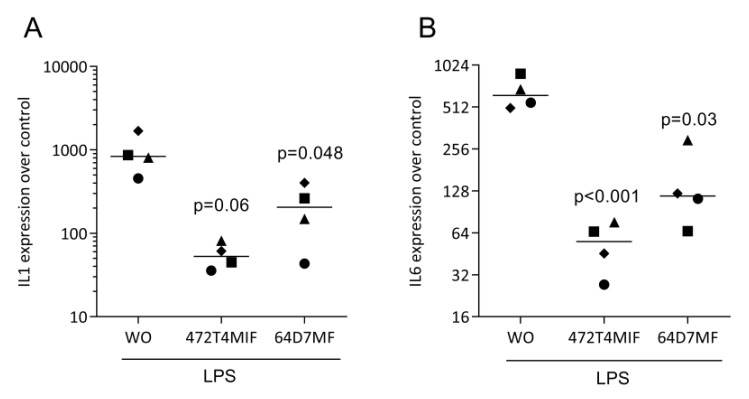
472T4MIF and 64D7MF suppress LPS-induced expression of cytokines in primary macrophages. Primary macrophages were exposed to 20 µM of 472T4MIF or 64D7MF for 30 min before cells were treated with 100 ng/mL LPS for 24 h. Data show the x-fold changes in (**A**) IL1 and (**B**) IL6 expression compared to the LPS group (WO: without). Statistical analysis was based on repeated measures one-way ANOVA with Greenhouse–Geisser correction and Dunnett’s multiple comparison test. The *p*-values are indicated compared to the stimulated group but without the flavonoids. Significance was set at *p <* 0.05.

To identify the possible activity, we have pre-exposed RAW 264.7 and primary macrophages to the two flavonoids before provoking an inflammatory response with LPS [27,28]. We found that the strong LPS increasedIL1 and IL6 expression was virtually eliminated in the presence of 20 µM 472T4MIF or 64D7MF in RAW 264.7 cells (Figure 3) and in primary macrophages (Figure 4). Immunoassays for IL6 confirmed that 472T4MIF and 64D7MF are capable to lower the LPS-induced production of IL6 by RAW 264.7 cells (Figure 3D). Additionally, CCL2 was also downregulated by 472T4MIF and 64D7MF in RAW 264.7 cells (Figure 3C). Overall, 472T4MIF was more potent compared to 64D7MF to reduce the inflammatory response of macrophages to LPS.

### 3.4. 472T4MIF and 64D7MF Diminished the p65 and p38 Phosphorylation in Macrophages

**Figure 5:**
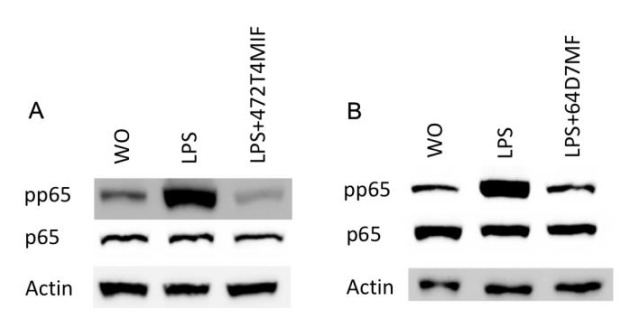
472T4MIF and 64D7MF reduced the phosphorylation of p65 in RAW 264.7 cells. RAW 264.7 cells were exposed to 20 µM of 472T4MIF or 64D7MF for 30 min before cells were treated with LPS for 30min. Western blot analysis shows the phosphorylation of p65 of cells stimulated with LPS but not in the untreated cells or in the presence of flavonoids.

**Figure 6:**
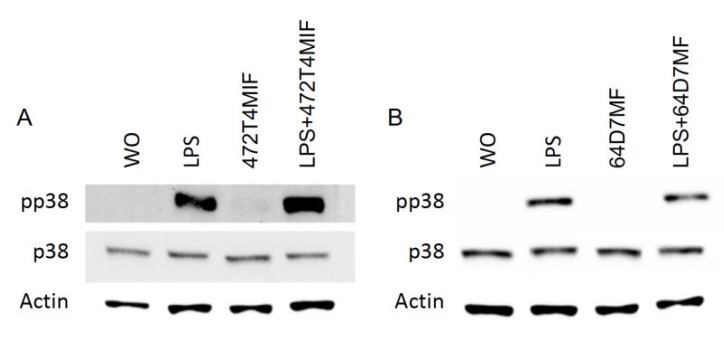
472T4MIF and 64D7MF failed to considerably reduce the phosphorylation of p38 in RAW 264.7 cells. RAW 264.7 cells were exposed to 20 µM of 472T4MIF or64D7MF for 30 min, then LPS for 30 min. Western blot analysis shows the phosphorylation of p38 of cells stimulated with LPS but not in the untreated cells or in the presence of flavonoids.

To identify the underlying molecular mechanism, we tested to which extent the flavonoids reduce the canonical p65-NFkB signalling pathway in RAW 264.7 macrophages. Western blot showed that the exposure of RAW 264.7 macrophages to 20 µM of 472T4MIF (Figure 5A) or 64D7MF (Figure 5B) greatly reduced the LPS-induced phosphorylation signal of p65. To detect the underlying molecular mechanism, we tested to which extent the flavonoids reduce the phosphorylation p38 in RAW 264.7 macrophages. Western blot showed that the exposure of RAW 264.7 macrophages to 20 µM of 472T4MIF (Figure 6A) or 64D7MF (Figure 6B) greatly reduced the LPS-induced phosphorylation signal of p38.Taken together, the activity of 472T4MIF and 64D7MF was associated with a significant blocking of p65 and p38 phosphorylation in vitro.

### 3.5. 472T4MIF and 64D7MF Do Not Reduce the Response of Fibroblasts to IL1β and TNFa

**Figure 7:**
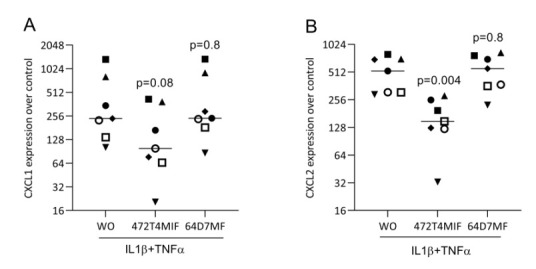
472T4MIFcan reduce CXCL1 and CXCL2 expression in gingival fibroblasts. Gingival fibroblasts were exposed to 20 µM of 472T4MIF or64D7MFfor 30 min before cells were treated with 10 ng/ml IL1β and TNFα for 24 h. Without (WO) indicates cells treated with IL1β and TNFα but no flavonoids. Data show the x-fold changes in (**A**) CXCL1 and (**B**) CXCL2 expression compared to unstimulated cells. Statistical analysis was based on repeated measures one-way ANOVA with Greenhouse–Geisser correction and Dunnett’s multiple comparison test. The *p*-values are indicated compared to the IL1β and TNFα group but without the flavonoids.

**Figure 8:**
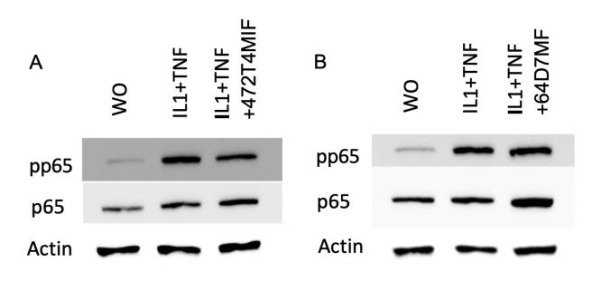
472T4MIF moderately reduce the phosphorylation of p65 in gingival fibroblasts. Gingival fibroblasts were exposed to 20 µM of 472T4MIF or 64D7MF before cells were treated with LPS for 30 min. Western blot analysis shows the phosphorylation of p65 of cells stimulated with IL1β and TNFα but not in the untreated cells. 472T4MIF (**A**), but not 64D7MF (**B**), caused a weak reduction in phosphorylation of p65.

Next, we determined if 472T4MIF and 64D7MF can lower the inflammatory response of gingival fibroblasts exposed to IL1β and TNFα. The gingival fibroblasts showed the expected strong inflammatory reactions expressed by the increased expression of CXCL1 and CXCL2 (Figure 7). Gingival fibroblasts responded to 472T4MIF but not to 64D7MF by a decrease in the inflammatory chemokines CXCL1 and CXCL2 (Figure 7). Nevertheless, Western blot analysis showed that 472T4MIF, but not 64D7MF, slightly lowered the p65 phosphorylation signal of gingival fibroblasts exposed to IL1β and TNFα (Figure 8).

## 4. Discussion

Herbal medicine was successful in supporting people’s health for centuries, and it is only recently that the pharmacological compounds that account for the beneficial effects are beginning to be identified. Complex extraction processes of plant-derived single molecules are followed by deciphering the chemical structure. It then requires the bioassay to identify the function of the single molecules that, depending on the purpose, can be a macrophages assay that simulates an inflammatory response. Macrophages are suitable model systems as they play a key role in the initiation of inflammation and its resolution in various diseases [29,30]. Taking advantage of this bioassay, we could identify two flavonoids isolated from the heartwood of *D. cochinchinensis*, namely 4,7,2′-trihydroxy-4′-methoxyisoflavanol and 6,4′-dihydroxy-7-methoxyflavane to greatly suppress the LPS-induced cytokine and CCL2 expression of murine macrophages. Moreover, 472T4MIF reduced the expression of CXCL1 and CXCL2 in gingival fibroblasts. The importance of this research was thus that we could add two more plant-derived molecules to the growing list of possible anti-inflammatory molecules. 

If we relate the findings observed with *D. cochinchinensis* to those of others, we can build up on the knowledge gained by *D. odorifera* and its phytocompounds showing activity in a similar model, the LPS-stimulated RAW 264.7 cells [11,12,16,19]. In general, *D. odorifera* species are widely used as medicinal drugs. Its approximately 175 metabolites are not only tested for bioactivity; however, they play an important role in drug discovery programs [31,32]. Based on similar strategies, we recently showed that *D.melanoxylon* contains 3′-hydroxy-4,4′-dimethoxydalbergione, 4-methoxydalbergione, and 4′-hydroxy-4-methoxydalbergione, all of which are inhibitors of LPS-stimulated macrophages in vitro (Shao et al. submitted). This progress in the phytochemistry and pharmacology of metabolites originating from *Dalbergia* species was not only to explain the traditional use but also for drug discovery. 

We further observed that 472T4MIF, but not 64D7MF, was capable to lower the IL1β and TNFα-induced expression of CXCL1 and CXCL2 by human gingival fibroblast. Consistently, when using fibroblast-like synoviocytes, for instance, neohesperidin [33], diosmetin [34], and kaempferitrin [35], all flavonoids, suppressed in vitro inflammation. Quercetin, a natural flavanol that reduced inflammatory cytokines [36], and luteolin reduced COX2 expression [37] in human gingival fibroblasts. This demonstrates that fibroblasts are potential targets for the activity of phytocompounds. In all studies mentioned, the cells were exposed to LPS. We, however, used IL1β and TNFα to provoke a robust inflammatory response in gingival fibroblasts as the 100 ng/mL LPS gave no robust in vitro inflammatory response. The findings thus suggest that at least 472T4MIF can lower the expression of chemokines induced by the IL1β and TNFα signalling pathway, but the reduction in phosphorylation of p65 was not substantial and presumably only partially dependent on NFkB signalling. 

The study has limitations. First, the screening for a possible anti-inflammatory activity remains restricted to an in vitro bioassay, therefore future studies should translate the findings to a preclinical situation, including models for rheumatoid arthritis and periodontitis. For instance, the flavonoids can be tested for their activity to reduce periodontal tissue destruction upon placing ligatures around teeth [38]. Secondly, the molecular mechanism that accounts responsible for the activity of 472T4MIF and 64D7MF in the macrophages has not been identified, but it obviously involves the NFkB signalling cascade as the phosphorylation of p65 was greatly suppressed. Surprisingly, however, we could not identify an expression change in the heme oxygenase-1 pathway that is linked to the activity of various phytocompounds (data not shown). For example, 4,2′,5′-trihydroxy-4′-methoxychalcone from the heartwood of *D. odorifera* [39] but also latifolin [40] induced heme oxygenase-1 expression through the nuclear translocation of nuclear factor E2-related factor 2 in macrophages. Hence, further research is required to understand how 472T4MIF and 64D7MF exert their activity on the molecular level including the question of how the effects restricted to TLR mediated signalling. Third, our in vitro models do not simulate inflammation derived from metabolic stress, and a short time exposure is not enough for the polarization of macrophages to develop an M1-like or M2-like phenotype. Hence future studies should consider the impact of the flavonoids on macrophage polarization and how this is affected by metabolic stress [41]. Finally, we are aware that *D. cochinchinensis* is engendered in the IUCN Red List of Threatened Species™; this research is not intended to support the traditional use of plant extracts but to use those to refine pharmacological drug discovery. 

In summary, we showed here that 4,7,2′-trihydroxy-4′-methoxyisoflavanol and 6,4′-dihydroxy-7-methoxyflavane can be isolated from the heartwood of *D. cochinchinensis*, both being inhibitors of an inflammatory response of murine macrophages exposed to LPS. We also learned that both phytocompounds failed to reduce the expression of inflammatory cytokines when exposed to IL1β and TNFα. These results add two new molecules to the list of phytocompounds with an LPS-induced activity.

## Figures and Tables

**Table 1:** The primer sequences.

mIL1β	AAGGGCTGCTTCCAAACCTTTGAC	ATACTGCCTGCCTGAAGCTCTTGT
mIL6	GCTACCAAACTGGATATAATCAGGA	CCAGGTAGCTATGGTACTCCAGAA
mCCL2	GCTACAAGAGGATCACCAGCAG	GTCTGGACCCATTCCTTCTTGG
mGAPDH	AACTTTGGCATTGTCGAACG	GGATGCAGGGATGATGTTCT
hCXCL1	TCCTGCATCCCCCATAGTTA	CTTCAGGAACAGCCACCAGT
hCXCL2	CCCATGGTTAAGAAAATCATCG	CTTCAGGAACAGCCACCAAT
hGAPDH	AAGCCACATCGCTC AGACAC	GCCCAATACGACCAAATCC

## Data Availability

Not applicable.

## Sample Availability

Samples of the compounds are available from the authors.
